# High Contagiousness and Rapid Spread of Severe Acute Respiratory Syndrome Coronavirus 2

**DOI:** 10.3201/eid2607.200282

**Published:** 2020-07

**Authors:** Steven Sanche, Yen Ting Lin, Chonggang Xu, Ethan Romero-Severson, Nick Hengartner, Ruian Ke

**Affiliations:** Los Alamos National Laboratory, Los Alamos, New Mexico, USA

**Keywords:** COVID-19, 2019 novel coronavirus disease, SARS-CoV-2, severe acute respiratory syndrome coronavirus 2, viruses, respiratory infections, zoonoses, Wuhan, China, transmission potential, modeling

## Abstract

Severe acute respiratory syndrome coronavirus 2 is the causative agent of the ongoing coronavirus disease pandemic. Initial estimates of the early dynamics of the outbreak in Wuhan, China, suggested a doubling time of the number of infected persons of 6–7 days and a basic reproductive number (R_0_) of 2.2–2.7. We collected extensive individual case reports across China and estimated key epidemiologic parameters, including the incubation period (4.2 days). We then designed 2 mathematical modeling approaches to infer the outbreak dynamics in Wuhan by using high-resolution domestic travel and infection data. Results show that the doubling time early in the epidemic in Wuhan was 2.3–3.3 days. Assuming a serial interval of 6–9 days, we calculated a median R_0_ value of 5.7 (95% CI 3.8–8.9). We further show that active surveillance, contact tracing, quarantine, and early strong social distancing efforts are needed to stop transmission of the virus.

Severe acute respiratory syndrome coronavirus 2 (SARS-CoV-2) is the etiologic agent of the current rapidly growing outbreak of coronavirus disease (COVID-19), originating from the city of Wuhan, Hubei Province, China (*1*). Initially, 41 cases of “pneumonia of unknown etiology” were reported to the World Health Organization by the Wuhan Municipal Health Committee at the end of December 2019 (*2*). On January 8, 2020, the pathogen was identified (*1*), and human-to-human transmission was reported soon after. By January 21, most provinces of China had reported COVID-19 cases. By March 16, the outbreak had led to >170,000 total confirmed cases and >6,500 deaths globally. In a period of 3 months, an outbreak of apparent idiopathic pneumonia had become the COVID-19 pandemic.

Studying dynamics of a newly emerged and rapidly growing infectious disease outbreak, such as COVID-19, is important but challenging because of the limited amount of data available. In addition, unavailability of diagnostic reagents early in the outbreak, changes in surveillance intensity and case definitions, and overwhelmed healthcare systems confound estimates of the growth of the outbreak based on data. Initial estimates of the exponential growth rate of the outbreak were 0.1–0.14/day (a doubling time of 6–7 days), and a basic reproductive number (R_0_; defined as the average number of secondary cases attributable to infection by an index case after that case is introduced into a susceptible population) ranged from 2.2 to 2.7 (*1*,*3*–*5*). These estimates were based on 2 broad strategies. First, Li et al. used very early case count data in Wuhan before January 4 (*1*). However, case count data can be confounded by reservoir spillover events, stochasticities in the initial phase of the outbreak, and low surveillance intensity. The epidemic curve based on symptom onset after January 4 showed a very different growth rate (*6*). Second, inference was performed by using international flight data and infected persons reported outside of China (*3*–*5*). Because of the low numbers of persons traveling abroad compared with the total population size in Wuhan, this approach leads to substantial uncertainties (*7*,*8*). Inferences based on a low number of observations are prone to measurement error when data are incomplete or model assumptions are not fully justified; both conditions are common challenges associated with rapid and early outbreak analyses of a new pathogen.

We collected an expanded set of case reports across China on the basis of publicly available information, estimated key epidemiologic parameters, and provided a new estimate of the early epidemic growth rate and R_0_ in Wuhan. Our approaches are based on integration of high-resolution domestic travel data and early infection data reported in provinces other than Hubei to infer outbreak dynamics in Wuhan. They are designed to be less sensitive to biases and confounding factors in the data and model assumptions. Without directly using case confirmation data in Wuhan, we avoid the potential biases in reporting and case confirmation in Wuhan, whereas because of the high level of domestic travel before the Lunar New Year in China, inference based on these data minimizes uncertainties and risk for potential misspecifications and biases in data and model assumptions.

## Methods

### Methodologic Overview

We developed 2 modeling approaches to infer the growth rate of the outbreak in Wuhan from data from provinces other than Hubei. In the first model, the first arrival model, we computed the likelihood of the arrival times of the first known cases in provinces outside of Hubei as a function of the exponential growing population of infected persons in Wuhan before late January. This calculation involved using domestic travel data to compute the probability that an infected person traveled from Wuhan to a given province as a function of the unknown actual number of infected persons in Wuhan and the probability that they traveled. The timings of the arrivals of the first infected persons in different provinces would reflect the rate of the epidemic growth in Wuhan.

In the second model, the case count model, we accounted for the detection of additional persons who were infected in Wuhan and received their diagnoses in other provinces and explicitly modeled those persons by using a hybrid deterministic–stochastic SEIR (susceptible-exposed-infectious-recovered) model. We then fitted this model to new daily case count data reported outside Hubei Province during the period before substantial transmission occurred outside of the province.

By using data collected outside Hubei Province, we minimized the effect of changes in surveillance intensity. By the time cases were confirmed in provinces outside Hubei, all of the provinces of China had access to diagnostic kits and were engaging in active surveillance of travelers out of Wuhan (e.g., using temperatures detectors and digital data to identify infected persons [*9*]) as the outbreak unfolded. Furthermore, the healthcare systems outside Hubei were not yet overwhelmed with cases and were actively searching for the first positive case, leading to much lower bias in the reporting in each province compared with the time series of confirmed cases in Wuhan.

### Data

#### Individual Case Reports

We collected publicly available reports of 140 confirmed COVID-19 cases (mostly outside Hubei Province). These reports were published by the Chinese Centers for Disease Control and Prevention (China CDC) and provincial health commissions; accession dates were January 15–30, 2020 (Appendix 1 Table 1). Many of the individual reports were also published on the China CDC official website (http://www.chinacdc.cn/jkzt/crb/zl/szkb_11803) and the English version of the China CDC weekly bulletin (http://weekly.chinacdc.cn/news/TrackingtheEpidemic.htm). These reports include demographic information as well as epidemiologic information, including potential periods of infection, and dates of symptom onset, hospitalization, and case confirmation. Most of the health commissions in provinces and special municipalities documented and published detailed information of the first or the first few patients with confirmed COVID-19. As a result, a unique feature of this dataset includes case reports of many of the first or the first few persons who were confirmed to have SARS-CoV-2 virus infection in each province, where dates of departure from Wuhan were available.

#### Travel Data

We used the Baidu Migration server (https://qianxi.baidu.com) to estimate the number of daily travelers in and out of Wuhan (Appendix 1 Table 2). The server is an online platform summarizing mobile phone travel data hosted by Baidu Huiyan (https://huiyan.baidu.com).

#### Calculations of R_0_ and Effect of Intervention Strategies

We considered realistic distributions for the latent and infectious periods to calculate R_0_. We described the methods we used to calculate R_0_ and the effect of intervention strategies on the outbreak (Appendix 2).

## Results 

### Estimating Distributions of Epidemiologic Parameters

We first translated reports from documents or news reports published daily from the China CDC website and official websites of health commissions across provinces and special municipalities in China during January 15–30, 2020. Altogether, we collected 137 individual case reports from China and 3 additional case reports from outside of China (Appendix 1 Table 1).

**Figure 1:**
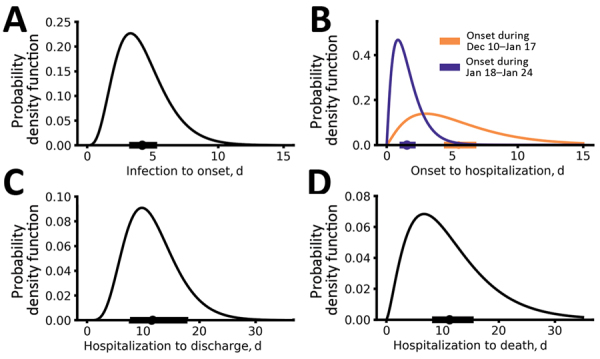
Epidemiologic characteristics of early dynamics of coronavirus disease outbreak in China. Distributions of key epidemiologic parameters: durations from infection to symptom onset (A), from symptom onset to hospitalization (B), from hospitalization to discharge (C), and from hospitalization to death (D). Filled circles and bars on *x*-axes denote the estimated means and 95% CIs.

By using this dataset, we estimated the basic parameter distributions of durations from initial exposure to symptom onset to hospitalization to discharge or death. Our estimate of the time from initial exposure to symptom onset (i.e., the incubation period) is 4.2 days (95% CI 3.5–5.1 days) (Figure 1, panel A), based on 24 case reports. This estimated duration is generally consistent with a recent report by Guan et al. (*10*) showing that the median incubation period is 4 days. Our estimate is ≈1 day shorter than 2 previous estimates (*1*,*11*). One potential caveat of our estimation is that because most of the case reports we collected were from the first few persons detected in each province, this estimation might be biased toward patients with more severe symptoms if they are more likely to be detected.

The time from symptom onset to hospitalization showed evidence of time dependence (Figure 1, panel B; Appendix 2 Figure 1). Before January 18, the time from symptom onset to hospitalization was 5.5 days (95% CI 4.6–6.6 days), whereas after January 18, the duration shortened significantly to 1.5 days (95% CI 1.2–1.9 days) (p<0.001 by Mann–Whitney U test). The change in the distribution coincides with news reports of potential human-to-human transmission and upgrading of emergency response level to Level 1 by the China CDC. The emerging consensus about the risk for COVID-19 probably led to substantial behavior changes among symptomatic persons, in terms of seeking more timely medical care during this period. However, because most of the individual reports were collected in provinces other than Hubei, the change in durations might only reflect changes in the rest of China (rather than in Hubei). We also found that the time from initial hospital admittance to discharge was 11.5 days (95% CI 8.0–17.3 days) (Figure 1, panel C) and from initial hospital admittance to death was 11.2 days (95% CI 8.7–14.9 days) (Figure 1, panel D). The time from symptom onset to death was estimated to be 16.1 days (95% CI 13.1–20.2 days).

### Estimating the Growth Rate of the Outbreak in Wuhan in January 2020

**Figure 2:**
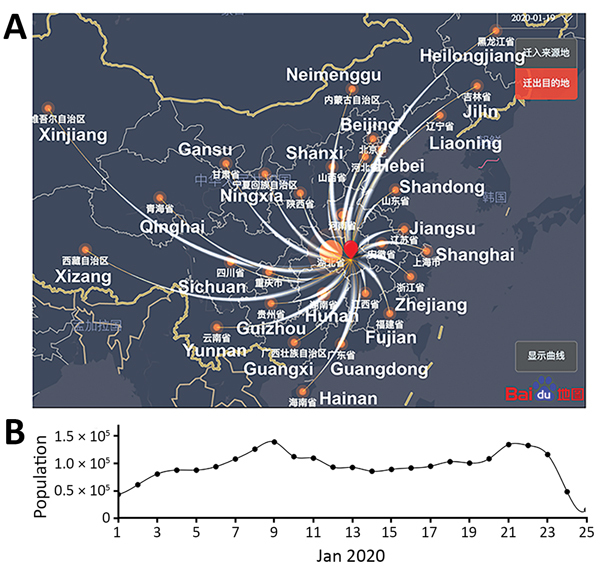
Extremely high level of travel from Wuhan, Hubei Province, to other provinces during January 2020, as estimated by using high-resolution and real-time travel data, China. A) A modified snapshot of the Baidu Migration online server interface showing the human migration pattern out of Wuhan (red dot) on January 19, 2020. Thickness of curved white lines denotes the size of the traveler population to each province. The names of most of the provinces are shown in white. B) Estimated daily population sizes of travelers from Wuhan to other provinces.

Moving from empirical estimates of basic epidemiologic parameters to an understanding of the early growth rates of COVID-19 requires model-based inference and data. We first collected real-time travel data during the epidemic by using the Baidu Migration server, which provides real-time travel patterns in China based on mobile-phone positioning services (Figure 2, panel A; Appendix 1 Methods Table 2,). We estimated that, before the January 23 lockdown of the city, ≈40,000–140,000 people in Wuhan traveled to destinations outside Hubei Province each day (Figure 2, panel B). The extensive travel before the Lunar New Year was probably an important driver of the spread of COVID-19 in China.

**Figure 3:**
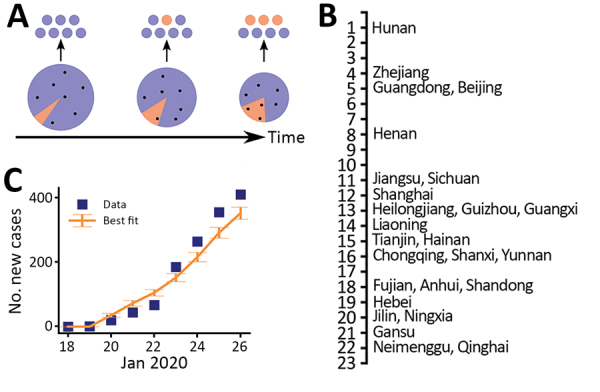
Estimates of the exponential growth rate and the date of exponential growth initiation of the 2019 novel coronavirus disease outbreak in China based on 2 different approaches. A) Schematic illustrating the export of infected persons from Wuhan. Travelers (dots) are assumed to be random samples from the total population (whole pie). Because of the growth of the infected population (orange pie) and the shrinking size of the total population in Wuhan over time, probability of infected persons traveling to other provinces increases (orange dots). B) The dates of documented first arrivals of infected persons in 26 provinces. C) Best fit of the case count model to daily counts of new cases (including only imported cases) in provinces other than Hubei. Error bars indicate SDs.

We then integrated spatiotemporal domestic travel data to infer the outbreak dynamics in Wuhan by using two mathematical approaches (Appendix 2; conceptual framework depicted in Figure 3, panel A). The first-arrival model uses a unique feature of our case report dataset whereby the dates of departure from Wuhan for many of the first persons who were confirmed with SARS-CoV-2 infection in each province were known (Appendix 1 Table 1). We assumed an exponential growth for the total infected population I* in Wuhan, , where I* includes infected persons who are asymptomatic or symptomatic, *r* is the exponential growth rate, and *t*_0_ is the theoretical time of the exponential growth initiation, so that I^*^(*t*_0_) = 1 in the deterministic model. We call *t*_0_ a “theoretical” time in the sense that it should not be interpreted as the time of first infection in a population. We should expect that *t*_0_ is later than the date of the first infection because multiple spillover events from the animal reservoir might be needed to establish sustained transmission and stochasticity might play a large role in initial dynamics before the onset of exponential growth (*12*–*14*).

We used travel data for each of the provinces (Appendix 1 Table 3) and the earliest times that an infected person arrived in a province, across a total of 26 provinces (Figure 3, panel B), to infer *r* and *t*_0_ (Appendix 2). Model predictions of arrival times in the 26 provinces fitted the actual data well (Appendix 2 Figure 2). The growth rate *r* is estimated to be 0.29/day (95% CI 0.21–0.37/day), corresponding to a doubling time of 2.4 days (95% CI 1.9–3.3 days). *t*_0_ is estimated to be December 20, 2019 (95% CI December 11–26). As we show later, there exist larger uncertainties in the estimation of *t*_0_.

We further estimated that the total infected population size in Wuhan was ≈4,100 (95% CI 2,423–6,178) on January 18 (Appendix 2 Figure 3), which is consistent with a recently posted estimate (*7*). The estimated number of infected persons was ≈18,700 (95% CI 7,147–38,663) on January 23 (i.e., the date when Wuhan started its lockdown). We projected that without any control measures, the infected population would be ≈233,400 (95% CI 38,757–778,278) by the end of January.

**Figure 4:**
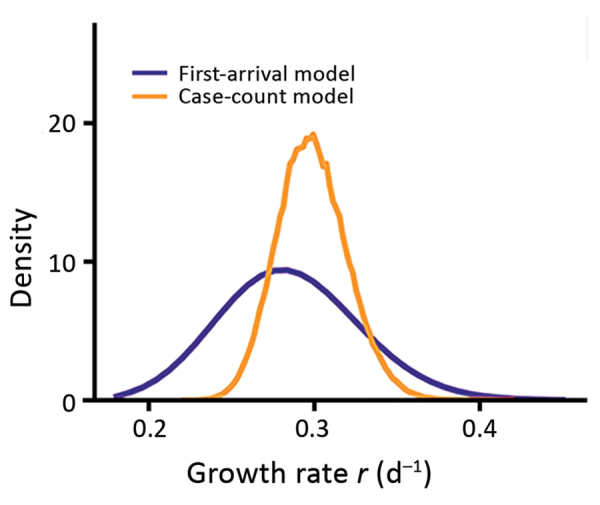
Marginalized likelihoods of growth rate (*r*) for 2 inference approaches to estimates the exponential growth rate of the coronavirus disease outbreak in China.

An alternative model, the case count approach, used daily new case counts of persons who had COVID-19 diagnosed in other provinces but who had been in Hubei Province within 14 days of becoming symptomatic. This model uses data beyond the first appearance of an infected person from Wuhan but also accounts for the stochastic nature of the process by using a hybrid model. In this model, the infected population in Wuhan was described with a deterministic model, whereas the infected persons who traveled from Wuhan to other provinces were tracked with a stochastic SEIR (susceptible-exposed-infectious-recovered) model (*12*). We restricted the data to the period of January 19–26, when new cases reported were mostly infections imported from Wuhan (i.e., indicative of the dynamics in Wuhan). The transitions of the infected persons from symptom onset to hospitalization and then to case confirmation were assumed to follow the distributions inferred from the case report data (Appendix 2). Simulation of the model using best-fit parameters showed that the model described the observed case counts over time well (Figure 3, panel C). The estimated theoretical time (*t*_0_) is December 16, 2019 (95% CI December 12–21), and the exponential growth rate is 0.30/day (95% CI 0.26–0.34/day). These estimates are consistent with estimates in the first arrival approach (Figure 4; Appendix 2 Figure 4).

In both models, we assumed perfect detection (i.e., of infected cases outside of Hubei Province). However, a certain fraction of cases probably was not reported. To investigate the robustness of our estimates, we performed extensive sensitivity analyses to test 23 different scenarios of surveillance intensity (Appendix 2). First, we tested the assumption that a constant fraction of infected persons (e.g., persons with mild or no symptoms) (*15*) were not detected. We found that under this assumption, *t*_0_would be earlier than our estimate but the estimation of the growth rate remained the same (Appendix 1 Table 4). Second, we tested the assumption that the intensity of surveillance increases over the period of data collection, although this scenario is less likely because of the intensive surveillance implemented outside Hubei Province. We found that our data in general do not support this hypothesis on the basis of corrected Akaike Information criterion scores (Appendix 1 Table 4). However, if the intensity of surveillance outside Hubei Province increased over the period of January, we would predict a lower growth rate than the estimate we just described. For the worst-case scenario considered, we estimated the growth rate of the outbreak to be 0.21/day (Appendix 2).

### Other Evidence of a High Growth Rate of the Outbreak in Wuhan

**Figure 5:**
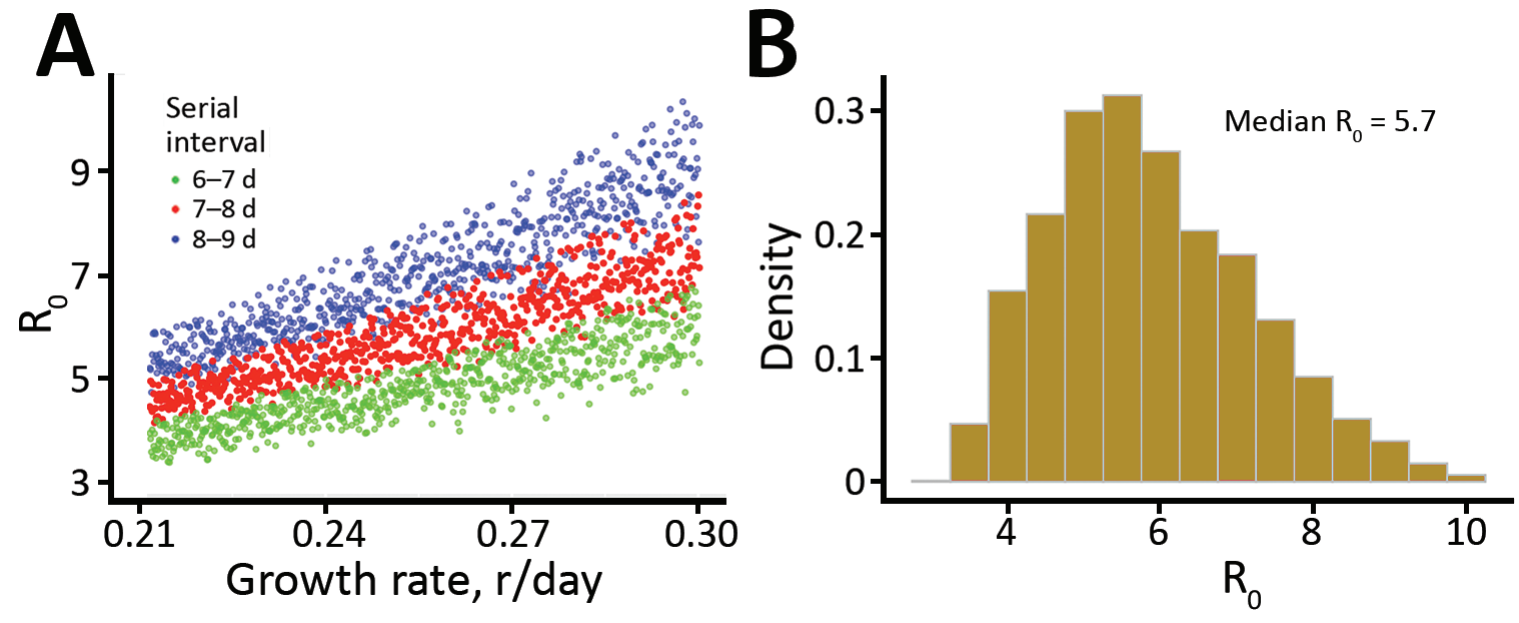
Estimation of the basic reproductive number (R_0_), derived by integrating uncertainties in parameter values, during the coronavirus disease outbreak in China. A) Changes in R_0_ based on different growth rates and serial intervals. Each dot represents a calculation with mean latent period (range 2.2–6 days) and mean infectious periods (range 4–14 days). Only those estimates falling within the range of serial intervals of interests were plotted. B) Histogram summarizing the estimated R_0_ of all dots in panel A (i.e., serial interval ranges of 6–9 days). The median R_0_ is 5.7 (95% CI 3.8–8.9).

In addition to using 2 modeling approaches, we looked for other evidence of a high outbreak growth rate to cross-validate our estimations. We found that the time series of reported deaths in Hubei, which is less subject to the biases of the confirmed case counts, is simply not consistent with a growth rate of 0.1/day (Appendix 2 Figure 5). As the infected population grows, the number of death cases will grow at the same rate but with a delayed onset corresponding to the time from infection to death. Fitting a simple exponential growth model to the number of reported deaths in Hubei during late January 2020 yields an estimate of 0.22–0.27/day, which is within the 95% CI of the estimation we previously described.

Overall, these analyses suggest that although there exist uncertainties depending on the level of surveillance, the exponential growth rate of the outbreak is probably 0.21–0.3/day. This estimation is much higher than previous reports, in which the growth rate was estimated to be 0.1–0.14/day (*1*,*3*–*5*).

### Estimating R_0_

**Figure 6:**
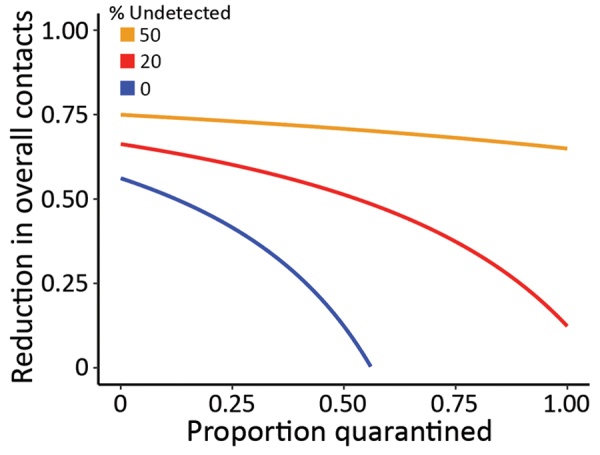
Levels of minimum efforts of intervention strategies needed to control the spread of severe acute respiratory syndrome coronavirus 2, (i.e. reducing the reproductive number to <1), during the coronavirus disease outbreak in China. Strategies considered were quarantine of infected persons and persons who had contact with them (*x*-axis) and population-level efforts to reduce overall contact rates (*y*-axis). Percentages denote the percentages of transmissions driven by infected persons that were not detected by surveillance as a result of asymptomatic infection, mild-to-moderate illness or low surveillance intensity.

The basic reproductive number, R_0_, is dependent on the exponential growth rate of an outbreak, as well as additional factors such as the latent period (the time from infection to infectiousness) and the infectious period (*16*,*17*), both of which cannot be estimated directly from the data. Following the approach by Wearing and Rohani (*16*), we found that with a high growth rate of the outbreak, R_0_ is in general high and the longer the latent and the infectious periods, the higher the estimated R_0_ (Appendix 2 Figure 6).

To derive realistic values of R_0_, we used previous estimates of serial intervals for COVID-19. The serial interval is estimated to be ≈7–8 days based on data collected early in the outbreak in Wuhan (*1*). More recent data collected in Shenzhen Province, China, suggests that the serial interval is dependent on the time to hospital isolation (Q. Bi et al., unpub. data, https://doi.org/10.1101/2020.03.03.20028423). When infected persons are isolated after 5 days of symptoms (a probable scenario for the early outbreak in Wuhan, where the public was not aware of the virus and few interventions were implemented), the serial interval is estimated to be 8 days (Q. Bi et al., unpub. data). Thus, these results suggest a serial interval of 7–8 days. With this serial interval, we sampled latent and infectious periods within wide biologically plausible ranges (Appendix 2) and estimated the median R_0_ to be 5.8 (95% CI 4.4–7.7) (Figure 5, panel A). To include a wider range of serial interval (i.e., 6–9 days) (Figure 5, panel A; Appendix 2 Figure 6), given the uncertainties in these estimations, we estimated that the median of estimated R_0_ is 5.7 (95% CI of 3.8–8.9) (Figure 5, panel B). The estimated R_0_ can be lower if the serial interval is shorter. However, recent studies reported that persons can be infectious for a long period, such as 1–3 weeks after symptom onset (*18*; R. Woelfel et al., unpub data. https://doi.org/10.1101/2020.03.05.20030502); thus, we believe that a mean serial interval shorter than 6 days is unlikely during the early outbreak in Wuhan, where infected persons were not rapidly hospitalized.

### Implications for Intervention Strategies

The R*_0_* values we estimated have important implications for predicting the effects of pharmaceutical and nonpharmaceutical interventions. For example, the threshold for combined vaccine efficacy and herd immunity needed for disease extinction is calculated as 1 – 1/R_0_. At R_0_ = 2.2, this threshold is only 55%. But at R_0_ = 5.7, this threshold rises to 82% (i.e., >82% of the population has to be immune, through either vaccination or prior infection, to achieve herd immunity to stop transmission).

We then evaluated the effectiveness for nonpharmaceutical interventions, such as contact tracing, quarantine, and social distancing, by using the framework by Lipsitch et al. (*19*) (Appendix 2). We extended the framework to consider a fraction of transmission occurring from infected persons who would not be identified by surveillance and can transmit effectively (*15*). This fraction is determined by the fraction of actual asymptomatic persons and the extent of surveillance efforts to identify these persons and persons with mild-to-moderate symptoms. Results show that quarantine and contact tracing of symptomatic persons can be effective when the fraction of unidentified persons is low. However, when 20% of transmission is driven by unidentified infected persons, high levels of social distancing efforts will be needed to contain the virus (Figure 6), highlighting the importance of early and effective surveillance, contact tracing, and quarantine. Future field, laboratory, and modeling studies aimed to address the unknowns, such as the fraction of asymptomatic persons, the extent of their transmissibility depending on symptom severity, the time when persons become infectious, and the existence of superspreaders are needed to accurately predict the impact of various control strategies (*20*).

## Discussion

In this study, we estimated several basic epidemiologic parameters, including the incubation period (4.2 days), a time dependent duration from symptom onset to hospitalization (changing from 5.5 days in early January to 1.5 days in late January outside Hubei Province), and the time from symptom onset to death (16.1 days). By using 2 distinct approaches, we estimated the growth rate of the early outbreak in Wuhan to be 0.21–0.30 per day (a doubling time of 2.3–3.3 days), suggesting a much faster rate of spread than initially measured. This finding would have important implications for forecasting epidemic trajectories and the effect on healthcare systems as well as for evaluating the effectiveness of intervention strategies.

We found R_0_ is likely to be 5.7 given our current state of knowledge, with a broad 95% CI (3.8–8.9). Among many factors, the lack of awareness of this new pathogen and the Lunar New Year travel and gathering in early and mid-January 2020 might or might not play a role in the high R_0_. A recent study based on structural analysis of the virus particles suggests SARS-CoV-2 has a much higher affinity to the receptor needed for cell entry than the 2003 SARS virus (*21*), providing a molecular basis for the high infectiousness of SARS-CoV-2.

How contagious SARS-CoV-2 is in other countries remains to be seen. Given the rapid rate of spread as seen in current outbreaks in Europe, we need to be aware of the difficulty of controlling SARS-CoV-2 once it establishes sustained human-to-human transmission in a new population (*20*). Our results suggest that a combination of control measures, including early and active surveillance, quarantine, and especially strong social distancing efforts, are needed to slow down or stop the spread of the virus. If these measures are not implemented early and strongly, the virus has the potential to spread rapidly and infect a large fraction of the population, overwhelming healthcare systems. Fortunately, the decline in newly confirmed cases in China and South Korea in March 2020 and the stably low incidences in Taiwan, Hong Kong, and Singapore strongly suggest that the spread of the virus can be contained with early and appropriate measures.

Appendix 1 Additional data for study of high contagiousness and rapid spread of severe acute respiratory syndrome coronavirus 2.

Appendix 2 Additional methods used for study of high contagiousness and rapid spread of severe acute respiratory syndrome coronavirus 2.
