# Supplementary material for: High Contagiousness and Rapid Spread of Severe Acute Respiratory Syndrome Coronavirus 2
Source: Emerg Infect Dis. 2020 Jul;26(7):1470–7. doi: 10.3201/eid2607.200282 (PMC7323562; doi:10.3201/eid2607.200282)
Supplement: Appendix 2 — Additional methods used for study of high contagiousness and rapid spread of severe acute respiratory syndrome coronavirus 2. [file 20-0282-Techapp-s2.pdf]

Article DOI: https://doi.org/10.3201/eid2607.200282

High Contagiousness and Rapid Spread of Severe Acute Respiratory Syndrome Coronavirus 2

Appendix 2

Travel Data

We used the Baidu Migration server (https://qianxi.baidu.com/) to estimate the number of daily travelers in and out Wuhan (Appendix 1 Table 2, https://wwwnc.cdc.gov/EID/article/26/7/20-0282-App1.xlsx). The server an online platform summarizing mobile phone travel data hosted by Baidu Huiyan (https://huiyan.baidu.com). Baidu Huiyan is a widely used positioning system in China. It processes >120 billion positioning requests daily through GPS, WIFI and other means (https://huiyan.baidu.com). Specifically, we extracted from the server the Immigration Index and Emigration Index for Wuhan based on cell phone positioning data. The indexes are linearly related to the number of travelers going in and out of Wuhan, respectively. We also extracted the fraction of individuals who went to or came from a particular province. It has been reported that there were 5 million people going out of Wuhan between January 10, i.e., the start of the Chinese New Year travel rush, and January 25 (https://www.washingtonpost.com/world/asia_pacific/china-coronavirus-live-updates/2020/01/30/1da6ea52-4302-11ea-b5fc-eefa848cde99_story.html; accessed Feb. 2, 2020). This allowed us to calibrate the Emigration Index and estimated the number of daily travelers to or from a particular province, and thus the fraction of people traveling to or from a particular province (Appendix 1 Table 3).

Estimating Distributions of Epidemiologic Parameters from Individual Case Reports

We used the first confirmed cases in provinces other than Hubei to inform the time between patient infection and the onset of symptoms ($n = 24$). These individuals had all traveled

to Wuhan a short time preceding symptoms onset. Since these individuals were the first cases detected in the province, it is likely that the infection occurred during their recent stay in Wuhan. We approximated the time of infection as the middle time point of their stay. Because the delays between infection and symptoms onset vary between patients, we modeled the delay using a gamma distribution, as its support is nonnegative and it permits relatively large delays as compared to the median. Figure 1 in the main text presents results from fitting the distribution to the data (https://wwwnc.cdc.gov/EID/article/26/7/20-0282-F1.htm).

The fitting procedure was performed by maximizing the likelihood of observed delays between infection and symptoms onset. For a single observation, the individual likelihood is the gamma density function evaluated at the infection-to-onset delay. Some of the delays were censored, i.e., bounded by a certain value. For example, in some cases, only the times of infection and hospitalization were reported, and the time of symptom onset was missing in the case report. In such cases, we assumed that the missing onset time is bounded between times of infection and hospitalization. Then, the likelihood for this observation is equal to the cumulative gamma distribution evaluated at this censored value, i.e., the time when the patient was hospitalized. The maximum likelihood estimates (MLEs) are the shape and scale parameters that maximize the sum over all observations of the individual log-likelihoods. We used differential evolution in scipy.optimize library (Python) to perform maximization. A stochastic algorithm was implemented in the optimization procedure to avoid being trapped in local minima (*1*). The likelihood-based confidence intervals was computed by methods reported in Raue et al (*2*).

A similar approach was adopted to fit distributions to the time between symptom onset and hospitalization ($n = 96$), between hospitalization and discharge ($n = 6$), and between hospitalization and death ($n = 23$). The reported dates for these events was obtained directly from official sources. Data from cases originating from all over China and neighboring countries were used for distribution fitting. Detailed patient-level data are provided in Appendix 1 Table 1.

The "First-Arrival" Model: Inferring Disease Dynamics in Wuhan Using the First-Arrival Times at Other Provinces

In this model, we used the first-arrival time of a patient who traveled from Wuhan to a specific other province and was later confirmed to have been infected by SARS-CoV-2. The

rationale behind our approach is that an increasing fraction of people infected in Wuhan increases the likelihood that one such case is exported to the other provinces. Hence, how soon new cases are observed in other provinces can inform the disease progression in Wuhan. We hypothesize that this information is more reliable because the infected population in Wuhan needs to sufficiently large to allow probable export of one infected individual. The flow of expected cases depends on the flow of travelers to each province and on the proportion of the Wuhan population that is infected by the virus.

We first estimated the daily number of travelers from Wuhan to each of the China provinces. For this purpose, we used Wuhan's daily migration index to other provinces and the daily distribution of traveler destinations from Wuhan (see Data Collection). When assuming linearity between the migration index and the total number of exported individuals, it can be estimated that a migration index of 1 is approximately equal to 5 million individuals over the sum of migration indexes from January 10 to January 25, 2020 (it was reported that 5 million individuals left Wuhan during that period; see Data Collection section). The total number of daily Wuhan travelers to a province at a certain date was then set equal to the number of travelers estimated from the migration index times the fraction of the population having traveled to this province. Results from estimation are reported in Appendix 1 Table 2.

An infected traveler may be pre-symptomatic, i.e., this individual may have been exposed to the virus (E) and not have developed symptoms or be already symptomatic (I). In fact, for many individuals, infection onset was recorded days after the time of their departure from Wuhan (see Appendix 1 Table 1). Assuming travelers represent a random sample of the whole population, it follows that the probability that a traveler is infected is equal to the number of exposed or infected individuals in Wuhan ($I^* = E + I$) over the total Wuhan population ($N(t)$). The total population size varied during the infection period. We estimated the population size by using the daily inflow and outflow of individuals from Wuhan (see Appendix 1 Table 2). To represent the beginning of an outbreak, we modeled an exponential increase in the size of exposed and infected population over time t:

$$I^*(t) = e^{r(t-t_0)} \qquad \text{(Equation 1)}$$

where r is the infection growth rate and t_0 is the time of onset of exponential outbreak.

Equation 1 allows a simple analytic expression of the likelihood of arrival times for the first cases in each of the provinces other than Hubei. For a specific province, indexed by i, we modeled the arrival of new cases in each province during short time intervals as a Poisson random process $X_t^{(i)}$. Note that the rate parameter of this Poisson process, $\lambda(t) = I^*(t)\,\kappa_i(t)/N(t)$ depends on the time-varying sum of exposed and symptomatic populations $I^*(t)$, the time varying flow of population $\kappa_i(t)$ transported from Wuhan to the province i and the time varying population size. It can be shown mathematically (*3*) that the probability that no exposed or symptomatic traveler arrived to province i during a short time interval $(t, t + \Delta t)$, $\Delta t \ll 1$ is:

$$\mathbb{P}\left\{X_{t+\Delta t}^{(i)} - X_t^{(i)} = 0\right\} \approx \exp\left(-\frac{I^*(t)\kappa_i(t)}{N(t)}\Delta t\right) \qquad \text{(Equation 2)}$$

We assume no delay was incurred due transportation in our model. Equation 2 is valid for any $t > 0$, and because the overall process is Markovian, we can formulate the probability that the time of arrival of the first case in province i, $T^{(i)}$, is later than t by:

$$\mathbb{P}\{T^{(i)} > t\} = \lim_{\Delta t \to 0} \prod_{j=1}^{M} \mathbb{P}\left\{X_{j\Delta t}^{(i)} - X_{(j-1)\Delta t}^{(i)} = 0\right\} = \exp\left(-\int_{t_0}^{t} \frac{I^*(s)\kappa_i(s)}{N(s)} ds\right) \qquad \text{(Equation 2)}$$

where $[t_0, t)$ was partitioned into M equal intervals of $\Delta t = j(t - t_0)/M$, and we convert the Riemannian sum into an integral in the limit of $M \to \infty$ ($\Delta t \to 0$). Finally, we apply $\mathrm{d}/\mathrm{d}t$ to $1 - \mathbb{P}\{T^{(i)} > t\}$ to obtain the probability density function (PDF) of the first-arrival time of province i:

$$\mathrm{PDF}_i(t) = \frac{I^*(t)\kappa_i(t)}{N(t)} \exp\left(-\int_{t_0}^{t} \frac{I^*(s)\kappa_i(s)}{N(s)} ds\right) \qquad \text{(Equation 3)}$$

The form of the probability density function Equation 4 was used to estimate the likelihood of observed arrival times in each province as a function of the growth rate r and outbreak initiation time t_0. This likelihood was maximized, again using differential_evolution in scipy.optimize (*1*), and the confidence intervals for r and t_0 were obtained through profile likelihood (*2*). Numerical integration was performed by discretizing time in daily time intervals, since both the flow of travelers and the population size in Wuhan were estimated daily.

Sensitivity Analyses for the "First-Arrival" Model

Under the 'first-arrival' model, it is assumed that all infected individuals since their arrival from Wuhan were eventually recorded/detected, i.e., 100% detection probability. However, it is possible that some first cases were missed by surveillance. Additionally, the model did not account for the possibility that detection efforts increased across provinces as the number of cases in Wuhan soared. Here, we perform sensitivity analyses to test the robustness of our estimation against these possibilities.

The model formulation above needed a small modification to perform here sensitivity analyses. The event Y: "no new arrival before time t is later diagnosed with the infection" is now equivalent to "no arrival of an infected individual before time t," "one infected arrival before time t remained undiagnosed," "two infected arrivals before time t remained undiagnosed," etc. For a Poisson process with fixed parameter λ, the probability of Y can be expressed as:

$$\mathbb{P}(Y) = \mathrm{e}^{-\lambda} + \sum_{k=1}^{\infty} \frac{(1-p)^k \lambda^k e^{-\lambda}}{k!} = e^{-\lambda p} \qquad \text{(Equation 4)}$$

where p is the probability of detection. It follows that the modified PDF formulation for sensitivity analyses is:

$$\mathrm{PDF}_i(t) = \frac{I^*(t)\kappa_i(t)\,p}{N(t)} \exp\left(-\int_{t_0}^{t} \frac{I^*(s)\kappa_i(s)\,p}{N(s)}\,ds\right) \qquad \text{(Equation 5)}$$

This PDF was used instead of equation 4 to obtain maximum likelihood estimates of the growth rate and outbreak initiation date for sensitivity analyses.

Results from Sensitivity Analyses

We evaluated the sensitivity of the growth rate estimate to these detection scenario uncertainties. A total of 23 detection scenarios were considered. As an illustration, Appendix 2 Figure 7 below describes two of these scenarios (purple and orange lines).

As shown in Appendix 2 Figure 7, we considered a start date of detection. We also considered the possibility that this date was December 25 or 31, which corresponds to the date of arrival of the first detected case in other provinces. After the start date, either the detection changed to a constant over time (the blue line), or the detection rate increases over time (the orange line). The detection probabilities were either constant over time (purple line) or increased

from early to late January (orange line). In scenarios with increasing detection over time, we considered that probabilities linearly increased either from 5% to 35%, or from 10% to 70%. The 7-fold increase was based on a recent paper from China CDC (*4*) showing that the case fatality ratio changes from 15% to 2% from early January to late January. This suggests a roughly 7-fold change in identifying infected individuals, assuming the true case fatality ratio shall be constant over time. Note that because this change reflects changes in Wuhan, rather than changes in non-Hubei provinces (from which data was used for inference), we think this 7-fold change is a maximal change given the high surveillance intensity outside of Hubei.

All considered scenarios along with their corresponding maximum likelihood estimate of the growth rate are reported in Appendix 1 Table 4. When the probability of detection was set to a constant level after the start date of detection (scenarios 1–12), the estimated growth rates are robust in the range of 0.28 to 0.29/day. t_0 changed in a wide range between Dec 3 and 21, 2019. When the probability of detection of a case was set to 10%, the estimated growth rate remained 0.29/day, but the estimated outbreak initiation date was Dec 12, 2019. When the probability of detection changes over time (scenarios 13–20), the estimated growth rates are in the range between 0.21–0.25/day.

An additional analysis we did was to fixed t_0 to December 1st and estimate detection levels (scenarios 21 to 23). Growth rate estimates in these cases are between 0.21 and 0.23/day, the detection level changed 2–3 folds.

Overall, growth rate estimates varied from 0.21 to 0.3 across scenarios. An evaluation of the AIC suggests that models with constant levels of detection better fit the data. This cannot be attributed to model parsimony as the number of estimated parameters was the same across all scenarios (two parameters). This could indicate relatively constant awareness in non-Hubei provinces where no or very few cases had been detected.

The "Case Count" Model: the SEIR-Type Hybrid Stochastic Model

Model 1 fitted the time of arrival of the first confirmed case of each province. We used a different approach and a different dataset to infer disease dynamics. In particular, we constructed a hybrid stochastic model for inferring the disease dynamics in Wuhan using daily counts of individuals who contracted the infection in Wuhan and were diagnosed outside Hubei province.

The model is hybrid in the sense that we will couple a deterministic and exponential growth to describe the outbreak in Wuhan and an agent-based model which describes the discrete population dynamics of the patients after they left Hubei to other provinces. In Appendix 2 Figure 8, we present a schematic diagram of the hybrid meta-population model.

Deterministic and Exponential Dynamics in Wuhan

We assume an exponential growth of the number of exposed (E_W, W for Wuhan) and symptomatic (I_W) populations in Wuhan over time, $E_W(t) = E_W(0)e^{rt}$ and $I_W(t) = I_W(0)e^{rt}$ from the onset. The overall growth rate r is dominated by the largest eigenvalue of a sequential compound process, and given an r value, the ratio $\phi \coloneqq E(0)/I(0)$ is asymptotic constant (*4*). Thus, given a growth rate parameter r and an initial condition $E(t_0) + I(t_0) = 1$, we numerically compute the exposed population $E(t) = \phi(r)\left(1 + \phi(r)\right)^{-1} \exp\left(r(t - t_0)\right)$ and the symptomatic population $I(t) = \left(1 + \phi(r)\right)^{-1} \exp\left(r(t - t_0)\right)$.

Agent-Based Model for Patients Who Have Left Wuhan to Other Provinces

We assume that between 1/1 and 1/26, the populations in Wuhan are large and the dynamics can be reasonably approximate by the above deterministic and exponentially growing curves. However, the initial propagation of the disease to other provinces in China involves only a small population of exposed (E_O, O for Others) or symptomatic individuals who left Hubei province. In addition, the transitions between different phases of these patients, from exposed (E_O) to symptomatic (I_O), over to hospitalized (H_O), and finally to be confirmed by laboratory examinations (C_O) in other provinces are also variable (as we quantified in Figure 1, panels C–F). Consequently, the resulting population dynamics in other provinces is highly stochastic. We thus adopt an agent-based modeling approach and rely on kinetic Monte Carlo Sampling techniques detailed below to simulate the population dynamics in other provinces. With this approach, we aim to generate samples of 1) each individual patient who left Wuhan at a specific date, and 2) the individual's health status as the time progresses (susceptible, exposed, or symptomatic). The goal is to accumulate a large amount of Monte Carlo samples, by which we can compute the key summary statistics, i.e., the average case reported on each day between 1/18 and 1/26, to be compared against to the data. We achieve this by the following algorithmic procedures.

1. Generate random number of infected populations leaving Wuhan. We collected migration index which quantifies the fraction of total populations (14 million) in Wuhan that

traveled to other provinces on each date $t_i = 1, \ldots, 26$ (see Appendix 1 Table 3). Assuming independence of an individual's health state (susceptible, exposed, or symptomatic) and the individual's migration decision (leaving to other provinces or not), on each date t_i, the exposed and symptomatic populations leaving Hubei can be modeled by two Binomial distributions, $B_E = \text{Binomial}\big(E_W(t_i), \mu(t_i)\big)$ and $B_I = \text{Binomial}\big(I_W(t_i), \mu(t_i)\big)$. Here, $E_W(t)$ and $I_W(t)$ are the exposed and symptomatic population in Wuhan, and are assigned to the nearest integers to the previously prescribed exponential growth, given model parameters (r, t_0). Thus, to generate one stochastic sample path (realization), we generate Binomially-distributed random populations leaving Hubei on each day between 1/1 and 1/26 (both included), and model each of these *in silico* patients' health states by the following procedures.

2. Generate the progression of the health state for each patient: We assume that each hypothetical patient generated by the above procedure would stochastically, identically and independently progress toward to be confirmed (C_O) and reported in one of the other provinces. If an individual was exposed (E_O) when s/he left Hubei at t_i, we generate a Gamma distributed random time $\Delta t_{E\to I} \sim \Gamma(\alpha_1, \beta_1)$ and update the individual's health state to symptomatic (I_O) at time $t_i + \Delta t_{E\to I}$. We chose a time-dependent waiting-time distribution for the progression from symptomatic sate I_O to reflect the two regimes we observed from the data (see main text): If $t_i + \Delta t_{E\to I}$ is before 1/18 (included), we generate a Gamma distributed random time $\Delta t_{I\to H} \sim \Gamma\big(\alpha_{2,1}, \beta_{2,1}\big)$ to model the waiting time for an infected patient to be hospitalized (otherwise, if it is later than 1/18, $\Delta t_{I\to H} \sim \Gamma\big(\alpha_{2,2}, \beta_{2,2}\big)$). Consequently, the patient's state is changed to H_O at time $t_i + \Delta t_{E\to I} + \Delta t_{I\to H}$. If $t_i + \Delta t_{E\to I} + \Delta t_{I\to H}$ is before 1/19, the patient would wait in the "H" state until 1/19 when the policy of case confirmation was announced and institutionalized. Then, the confirmation process is modeled by another Gamma distributed random time $\Delta t_{H\to C} \sim \Gamma(\alpha_3, \beta_3)$. The patient is then confirmed and reported at time $t_i + \Delta t_{E\to I} + \Delta t_{I\to H} + \Delta \mathrm{t}_{H\to C}$, and we add one more case report at the next integer (date of January). Similar procedure applied to a patient who had already progressed to the I_W state before s/he left Hubei on date t_i, with the exception that the first random waiting time is neglected—the patient's confirmation time would be $t_i + \Delta t_{I\to H} + \Delta_{H\to C}$. We repeat the procedure for each *in-silico* patient who left Wuhan between 1/1 and 1/26 (both included), and register the time when these patients were reported between 1/18 and 1/26 (both included).

Parameter Estimation and Uncertainty Quantification of (r, t_0)

It is our task to infer the unknown parameters, exponential growth rate r and exponential growth onset time t_0 by the number of confirmed cases reported between 1/18 and 1/26. This is possible because the information of the unknown parameters (r, t_0) have an impact of the deterministic growths of the exposed $E_W(t)$ and symptomatic population $I_W(t)$, which in turn have an impact on the random populations which have left Hubei on each date. These populations follow statistically quantified processes until the final confirmation outside of Hubei, and can be compared against the reported data.

An error measure is devised to assess the quality of fit of the model given a set of parameters (r, t_0) by the following procedures. For each parameter set, we generate $2^{13} = 8192$ Monte Carlo samples. On each date t_i, the jth sample reports a random number $n_C^{MC}(t\_i|r, t_0, j)$ of confirmed new cases. We thus average over all the samples and obtain an averaged number of newly confirmed cases on a date t_i, $n_C^{MC}(t_i|r, t_0) \coloneqq \sum_{j=1}^{8192} n_C^{MC}(t_i|r, t_0, j)$, and compare it to the actual data $n_C^{Data}(t_i)$. We quantify the quality of the fit by computing the sum of the squared residuals:

$$\varepsilon^2(r, t_0) \coloneqq \sum_{t_i=18}^{26} \left[n_C^{MC}(t_i|r, t_0) - n_C^{Data}(t_i)\right]^2 \qquad \text{(Equation 6)}$$

A 100×100 grid-based parameter scan is performed to identify the parameters in the region $0.22 < r < 0.42$ and $-20 \le t_0 \le -5$ for identifying the best-fit parameters:

$$r^*, t_0^* \coloneqq \text{argmin}_{\{r, t_0\}} \varepsilon^2(r, t_0) \qquad \text{(Equation 7)}$$

As for uncertainty quantification, we formulate the logarithm of the likelihood $\mathcal{L}$ of a parameter set (r, t_0) as

$$\log \mathcal{L}(\alpha, \tau) \coloneqq -n \frac{\varepsilon^2(r, t_0)}{\varepsilon^2(r^*, t_0^*)} \qquad \text{(Equation 8)}$$

Here, $n = 9$ is the number of data points we use to fit the model. The assumption we make to formulate the above likelihood is that 1) the data (number reported new cases on date t_i) is normally distributed with a mean which equals to the Monte Carlo mean reported new cases in

our model, and 2) the variance of the noise is identically and t_i-independently distributed, and the variance is equal to the mean squared residuals of the best-fit model.

We can then formulate a likelihood ratio test, which quantifies how likely a set of parameters (r, t_0) is in comparison to the best-fit parameters (r^*, t_0^*):

$$\mathbb{P}\{r, t_0 \mid Data\} \sim \exp\left[-n\left(1 - \frac{\varepsilon^2(r, t_0)}{\varepsilon^2(r^*, t_0^*)}\right)\right] \quad \text{(Equation 9)}$$

In Bayesian inference, what we computed is essentially the joint posterior distribution of the model parameters (r, t_0), provided a uniform prior distribution on the region of our interests. We present this joint distribution in Appendix 2 Figure 5. Finally, because the joint posterior is narrowly distributed, we can numerically compute the marginalized posterior,

$$\begin{aligned} \mathbb{P}\{r \mid Data\} &\sim \int \mathbb{P}\{r, t_0 \mid Data\}\, \mathrm{d}t_0 \\ \mathbb{P}\{t_0 \mid Data\} &\sim \int \mathbb{P}\{r, t_0 \mid Data\}\, \mathrm{d}r \end{aligned} \quad \text{(Equation 10)}$$

which is reported in Figure 4 and used to calculate the bounds of centered 95% probability mass to estimate the confidence interval of the growth rate r.

Calculation of *R*$_0$ from Estimated Exponential Growth Rates

Assuming gamma distributions for the latent and infectious periods, Wearing et al. (*5*) have shown that the value of R_0 can be calculated from estimated exponential growth rate, r, of an outbreak as:

$$R_0 = \frac{r\left(\frac{r}{\sigma m} + 1\right)^m}{\gamma\left[1 - \left(\frac{r}{\gamma n} + 1\right)^{-n}\right]} \quad \text{(Equation 12)}$$

where $1/\sigma$ and $1/\gamma$ are the mean latent and infectious periods, respectively, and m and n are the shape parameters for the gamma distributions for the mean latent and infectious periods, respectively.

To quantify the uncertainty of R_0, we assumed that m = 4.5 (same as the shape parameter we estimated for the incubation period), n = 3. The parameters (r, σ, γ) are assumed to be mutually independent and we generate random samples according to ranges of variations defined in Appendix 1 Table 5 to compute the resulting R_0. We generated 10^4 parameters, accepted those that result in a serial interval within the range of interests, and then computed their respective R_0 using Equation 12. We used the 97.5% and 2.5% percentile of the generate data to quantify the 95% confidence interval.

Calculation of the Impact of Intervention Strategies

Using a susceptible–exposed (noninfectious)– infectious–recovered (SEIR) type compartmental model, Lipsitch et al. (*6*) evaluated the impact of quarantine of symptomatic cases and their contacts to prevent further transmission. Assuming that only symptomatic individuals transmit the pathogen, they showed that the reproductive number after the intervention, R_{int}, can be expressed as:

$$R_{int} = \frac{R(1-q)D_{int}}{D} \qquad \text{(Equation 11)}$$

where R is the reproductive number before intervention, q is the percentage of infected individuals being quarantined, D_{int} and D are the mean durations of infectious period after intervention and without intervention, respectively.

Here in our model, we adopted this formulation; however, we assumed that a fraction, f, of infected individuals are asymptomatic and can transmit. In this case, quarantine of symptomatic individuals only reduces the contribution of these individuals toward the reproductive number. Thus, we can calculate the reproductive number under quarantine, R_q, as:

$$R_q = fR + (1-f)R_{int} = R\left(f + (1-f)(1-q)\frac{D_{int}}{D}\right) \qquad \text{(Equation 12)}$$

We also considered another form of control measure, i.e., the population-level control measure that reduces overall number of daily contacts in the population by ε. These measures include closing down of transportation systems, work and/or school closure, etc. Since R depends

linearly on the number of daily contacts, we calculate the combined impact of the individual-based quarantine and the population level control measure as:

$$R_{combine} = (1-\varepsilon)[fR + (1-f)R_{int}] = (1-\varepsilon)R\left(f + (1-f)(1-q)\frac{D_{int}}{D}\right) \quad \text{(Equation 13)}$$

In our calculations, we assume that the mean duration of infectious period of COVID-19 to be 10 days, i.e. D = 10 days. We further assume that intervention can reduce infectious period to 4 days, i.e., $D_{int} = 4$ days, based on data on the time from symptom onset to hospitalization from Singapore (*7*) and that individuals may transmit the virus before symptom onset. Since Singapore has one of the best surveillance systems for emerging infectious diseases like COVID-19, the value of D_{int} used here shall represent the best scenario for case isolation intervention. We set the value of R to be the median estimate of R_0, i.e., $R_0 = 5.7$.

References

1. Storn R, Price K. Differential evolution—a simple and efficient heuristic for global optimization over continuous spaces. J Glob Optim. 1997;11:341–59. https://doi.org/10.1023/A:1008202821328

2. Raue A, Kreutz C, Maiwald T, Bachmann J, Schilling M, Klingmüller U, et al. Structural and practical identifiability analysis of partially observed dynamical models by exploiting the profile likelihood. Bioinformatics. 2009;25:1923–9. PubMed https://doi.org/10.1093/bioinformatics/btp358

3. Cox DR, Oakes D. Analysis of survival data. Boca Raton (Florida): Chapman & Hall/CRC; 1984.

4. Novel Coronavirus Pneumonia Emergency Response Epidemiology Team. The epidemiological characteristics of an outbreak of 2019 novel coronavirus diseases (COVID-19) in China [in Chinese]. Zhonghua Liu Xing Bing Xue Za Zhi. 2020;41:145–51. PubMed

5. Wearing HJ, Rohani P, Keeling MJ. Appropriate models for the management of infectious diseases. PLoS Med. 2005;2:e174. PubMed https://doi.org/10.1371/journal.pmed.0020174

6. Lipsitch M, Cohen T, Cooper B, Robins JM, Ma S, James L, et al. Transmission dynamics and control of severe acute respiratory syndrome. Science. 2003;300:1966–70. PubMed https://doi.org/10.1126/science.1086616

7. Ng Y, Li Z, Chua YX, Chaw WL, Zhao Z, Er B, et al. Evaluation of the effectiveness of surveillance and containment measures for the first 100 patients with COVID-19 in Singapore—January 2–

February 29, 2020. MMWR Morb Mortal Wkly Rep. 2020;69:307–11. PubMed https://doi.org/10.15585/mmwr.mm6911e1

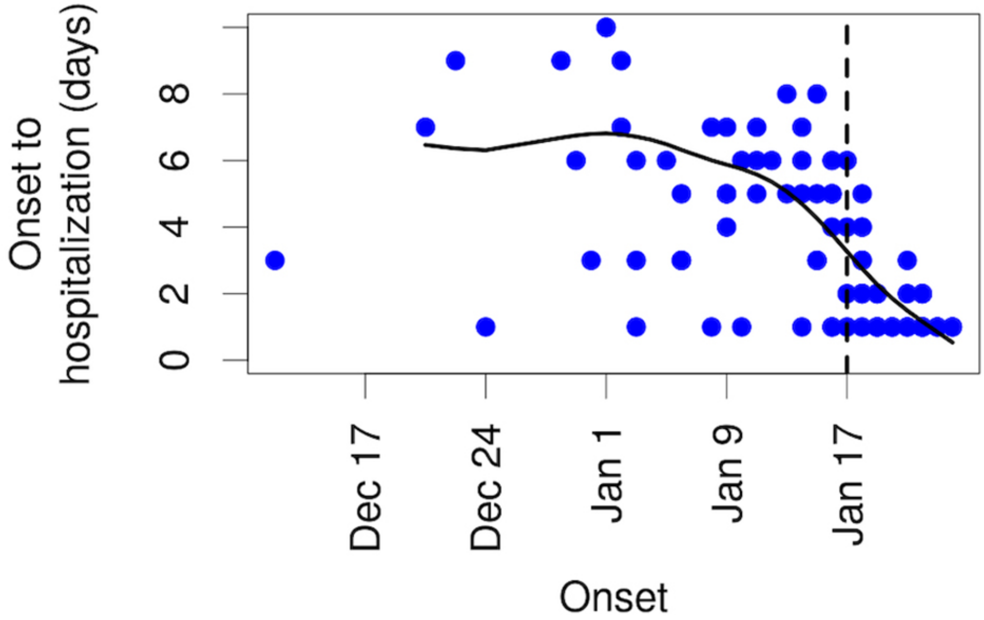

Appendix 2 Figure 1. The duration from symptom onset to hospitalization (y-axis) decreases over time during the outbreak.

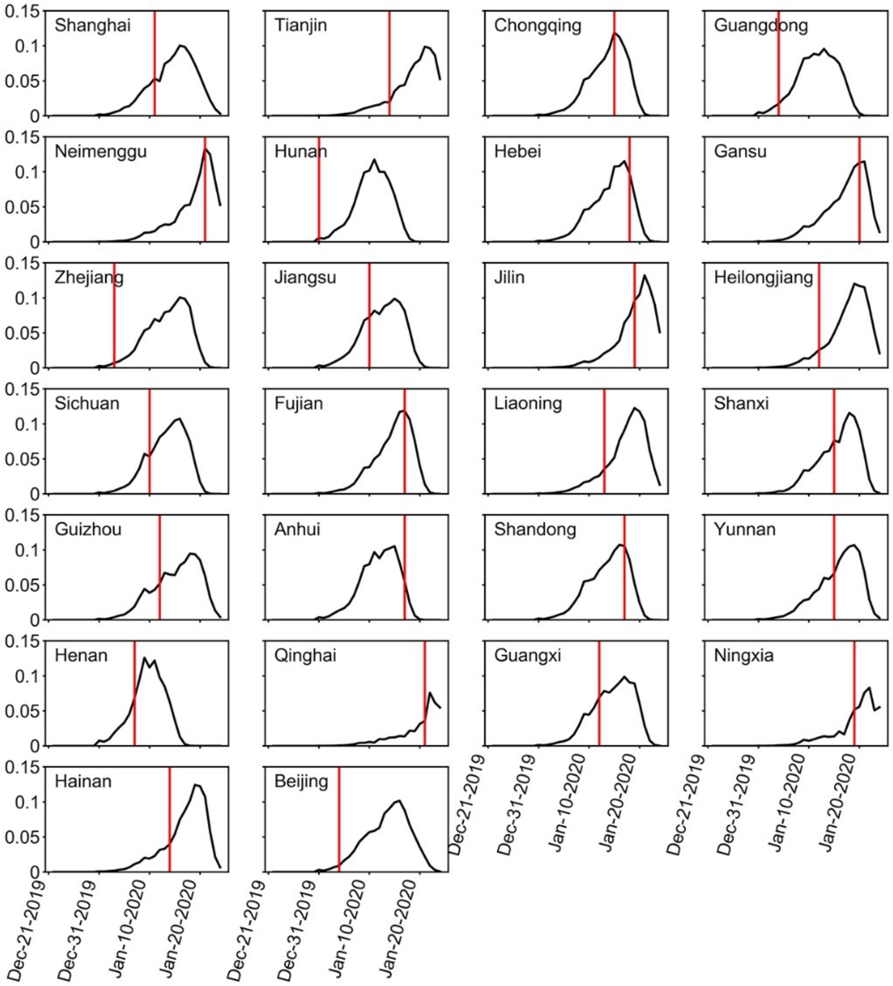

Appendix 2 Figure 2. Predictions of the ‘first arrival’ model using best-fit parameters agree well with data. Probability densities of times of first arrival of infected cases in each province based on our maximum likelihood estimate (curves) and documented times of first arrival of infected individuals in our case report dataset (lines).

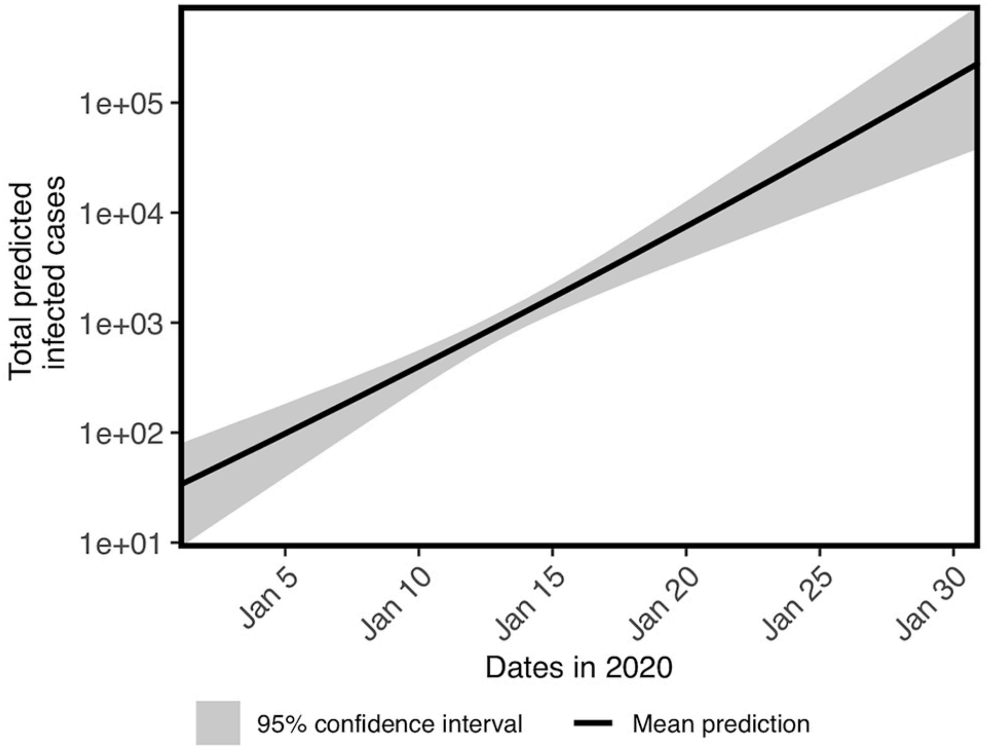

Appendix 2 Figure 3. Projections of numbers of infected individuals in Wuhan between January 1 and 30, 2020 using the likelihood profile of parameter values in the ‘first arrival’ approach. Projections after the lock-down of Wuhan on January 23 were hypothetical scenarios assuming no control measures are implemented.

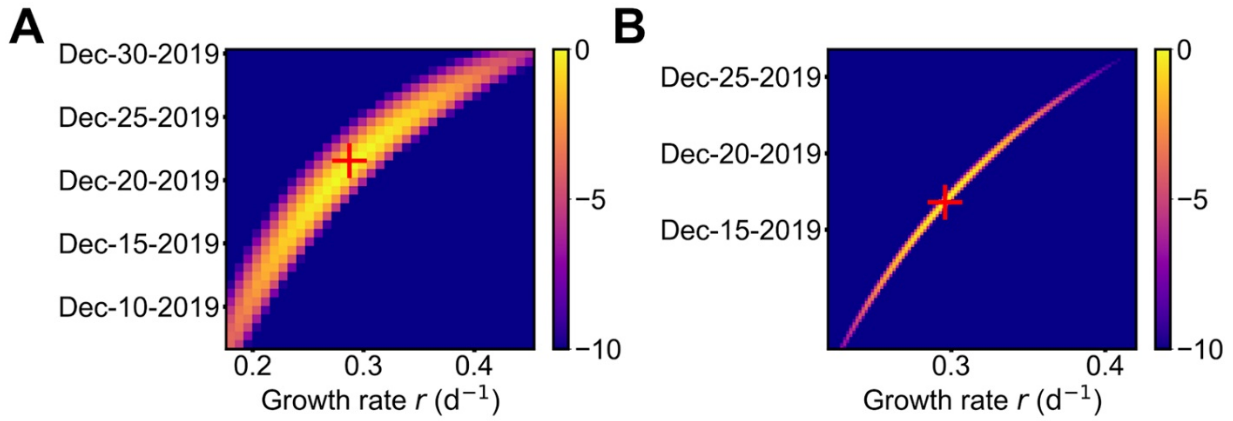

Appendix 2 Figure 4. Log-likelihood profiles of the estimated exponential growth rate of the outbreak, r (x-axis) and the date of exponential growth initiation (y-axis) from the ‘first arrival’ model (A) and the ‘case count’ model (B).

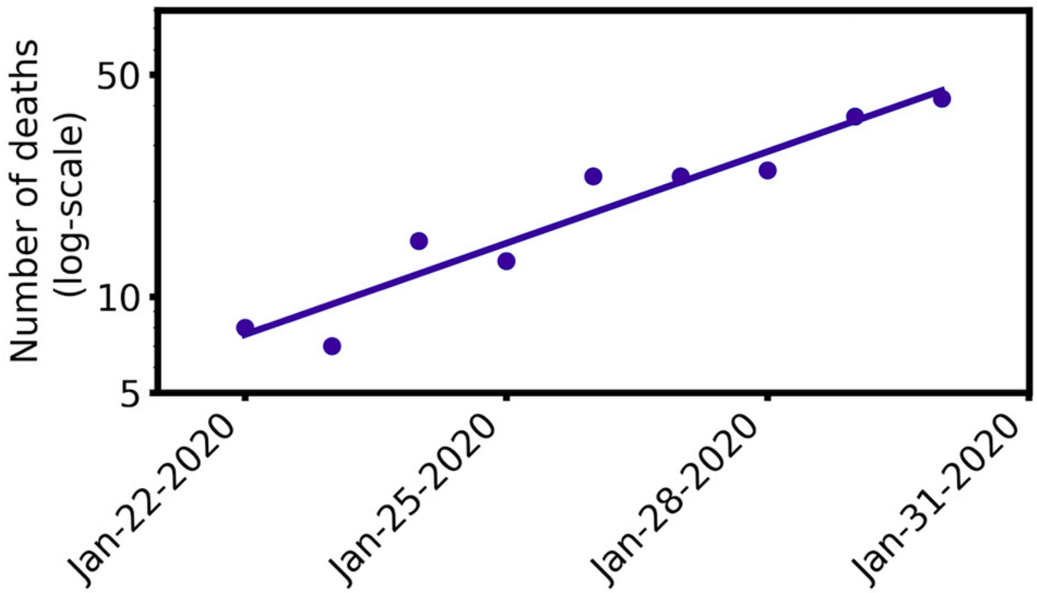

Appendix 2 Figure 5. The growth rate of the number of daily new death cases (on a log scale) in Hubei province in late January 2020 is estimated to be 0.27/day for cases collected between Jan. Twenty-two and 29, 2020. Dots and the blue line denote data and a fitted regression line, respectively. Note, there is a decrease in the growth rate after Jan 29, possibly reflecting intervention efforts or overwhelmed hospital system. When we include the data points on Jan. Thirty and 31, we get a growth rate of 0.22/day. We think the estimation using early data points are a better reflection of the early infection dynamics; however, we report a growth rate of the new death counts to be between 0.22 and 0.27/day.

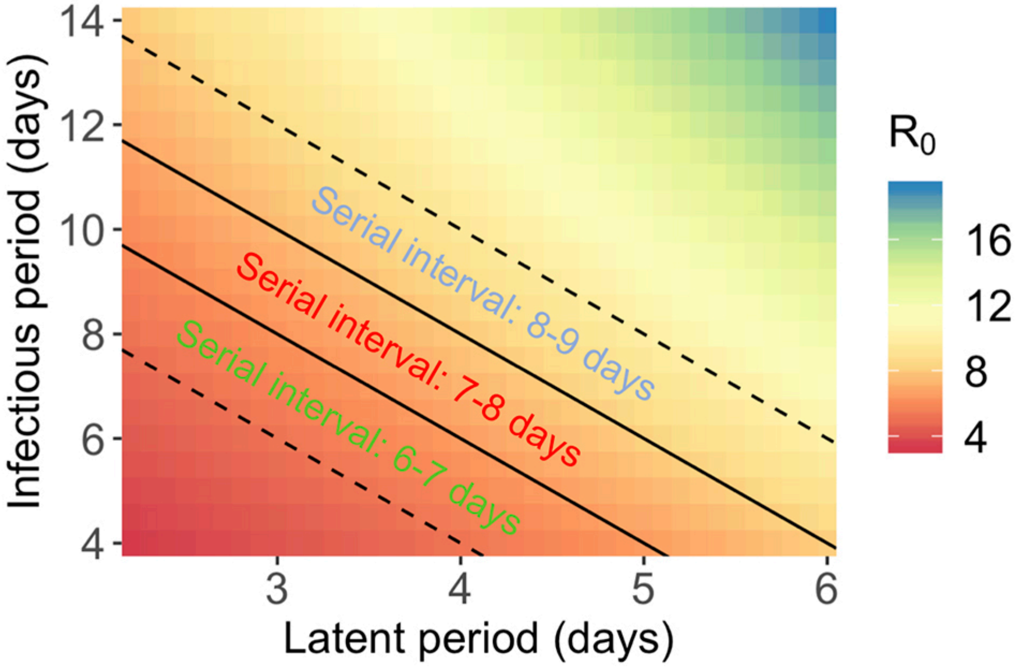

Appendix 2 Figure 6. Heatmap showing how R_0 changes with the mean durations of latent period and infectious period. The mean latent period is varied between 2.2 days and 6 days. The lower bound include the possibility that infected individual becomes infectious 2–3 days before symptom onset. The mean infectious period is varied between 4–14 days. The outbreak growth rate, r, is set to 0.29/day. Solid and dashed lines denote serial interval of 6, 7, 8 and 9 days, where we assumed the serial interval is the sum of the latent period and the half of the infectious period.

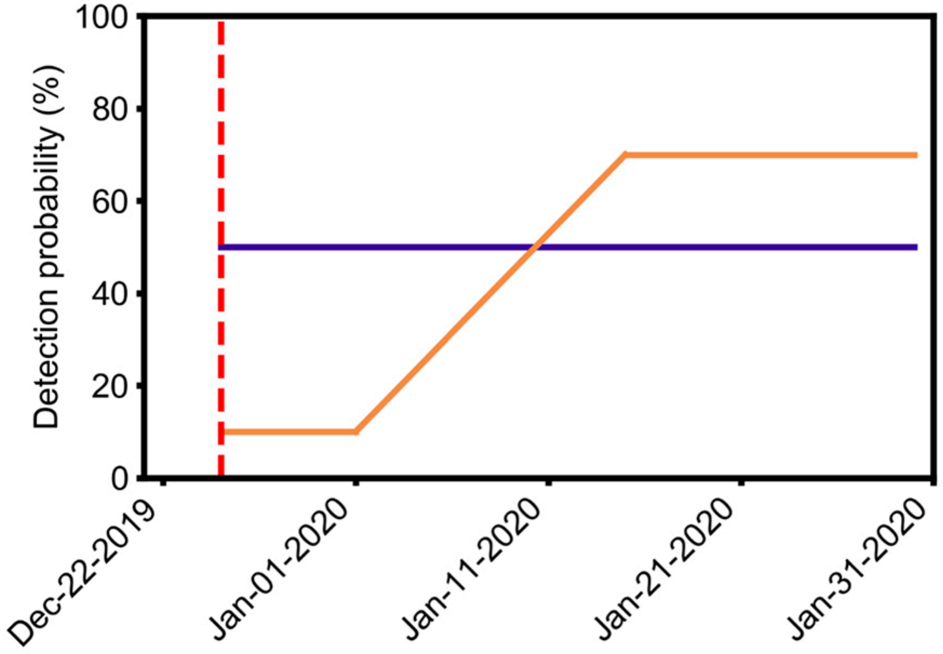

Appendix 2 Figure 7. Illustration of two detection scenarios (blue and orange lines) considered in sensitivity analysis. In both illustrated scenarios, it was considered impossible that a case who had arrived from Wuhan before Dec. 25, 2019 could be later detected with coronavirus (red dashed line), i.e., 0% detection before Dec 25. In the blue scenario, the detection probability changes from 0 to 50% after Dec 25; whereas the detection probability changes from 0 to 10% after Dec 25 and increases from 10% to 70% linearly between Jan. One and 15, 2020.

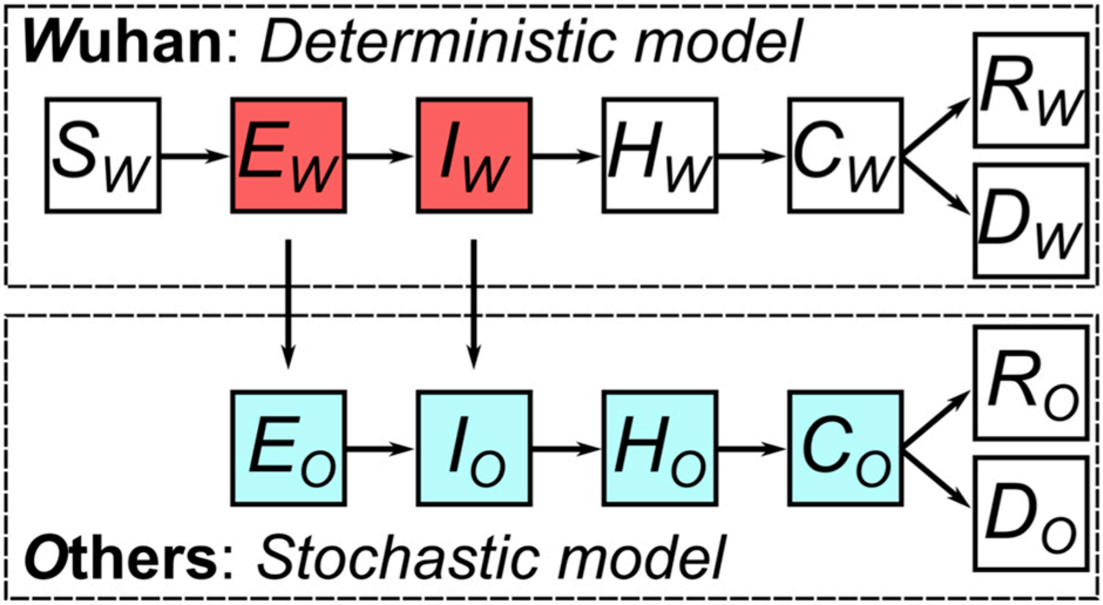

Appendix 2 Figure 8. Schematic diagram of the proposed meta-population model. Schematic diagram of the hybrid stochastic model. The model is a variant of the SEIR model with two geographic compartment, Wuhan (subscripted W) and other provinces (subscripted O). In Wuhan, a susceptible patient in compartment S_W is first exposed and progresses to an exposed state (E_W), progressed to be infected (I_W), hospitalized (H_W), and then became a confirmed case (C_W), and either recovered (R_W) or deceased (D_W). A portion of ill population (E_W and I_W) moved to other provinces and followed a similar progression. Because these populations are small and thus the dynamics are stochastic, we adopt an agent based approach to simulate the disease dynamics ($E_O(t)$, $I_O(t)$, $H_O(t)$ and $C_O(t)$) in other provinces. The case reports on each day in other provinces were compared against the model's output, $C_O(t)$ to constrain the unknown initial onset and growth rate in Wuhan.
